# The Bacteria of the Mouth—Of Interest to Dentists and Others

**Published:** 1892-07

**Authors:** Frank Jay Thornbury

**Affiliations:** 610 Main Street; Buffalo, N.Y.


### THE BACTERIA OF THE MOUTH.—OF INTEREST TO DENTISTS AND OTHERS.

By FRANK JAY THORNBURY, M. D., Buffalo, N. Y.

The oral cavity is a favorite location for various forms of micro-organisms. And it is not surprising that this should be the case when we consider the variety of circumstances conducive to bacteria gaining entrance to the mouth.

The principal sources for such access of germs are: Water, uncooked food, air, and certain other causes, active especially in children.

Secondly, we have here all the factors conducive to the growth and proliferation of microbes. There is continued moisture and a uniform temperature, and carbo-hydrates, and albuminous substances, which form the best possible nutrient media for bacteria.

These substances are almost invariably present between the teeth, in the cavities and depressions, and on the gums.

Over 100 different species of bacteria have been detected in the sputum. Many of these have only a transitory presence, but there are certain others constant in the mouth, and whose individual characteristics are now well understood. To Miller are we especially indebted for placing the subject of dental bacteriology on its present advanced basis. To refer to a few of the better known *mouth bacteria*, we would mention (1) Leptothrix innominata as occupying the foremost place in order of importance. It is by reason of its wonderful destructive properties that this bacillus has attracted so *much* attention. It stands in direct causative relation to many diseases of the buccal cavity, and has been known to penetrate the lungs, stomach, and other structures. The teeth themselves are sometimes permeated and disintegrated by this virulent microbe. It occurs in long thin threads, assuming a spiral form, and is found in soft white deposits on teeth in every mouth. With a solution of iodine and iodide of potassium it stains very readily; (2) Bacillus bucallis maximus is second in importance of the mouth bacteria, and occurs in aggregated tufts. Occasionally the individual threads are seen, and sometimes the bacilli themselves; (3) Leptothrix bucallis maxima.—This occurs as long thick filaments, sometimes curved, and is found in mucous deposits upon the teeth; (4) Jodoccus vaginatus.—Occurs singly or in chains of from two to ten cells encapsulated and is found in all unclean mouths; (5) Spirillum sputgenum, is sometimes rod shaped, but is usually of comma form, and has an active spiral movement. It is found in all mouths, especially where cleanliness is neglected. It is permissible of a staining with iodine solution ; (6) Spirochete dentium forms irregular threads of unequal thickness, and is found under the margin of the gums, when covered with a dirty deposit, or on gums in state of slight inflammation, *i. e.*, gingivitis marginalis.

Linsley has cultivated many additional varieties from the teeth and gums of patients suffering from lesions of the mouth, cases referred to him by dentists for bacteriological investigation. A particle of the suspected material is removed with a sterilized needle and placed in a gelatine or ager tube, after which the cultivation is easily affected.

The organic and inorganic substances found in the mouth, which may be the nidus of bacteria, are : (1) The normal saliva ; (2) Buccial mucus ; (3) Dead epithelium ; (4) Dental tissue softened by acids ; (5) Exposed pulp ; (6) Tartar and exudations on the gums ; (7) Accumulation of particles of food. There are three or four chromogenic or color-producing bacteria in the buccial cavity ; they give a blue or violet reaction with iodine, and form twenty to forty cultivable sub-species, partially non-pathogenic, partially of unknown pathogenesis.

The following pathogenic bacteria have been isolated from the mouth : (1) Micrococcus gingive pyogenes ; (2) Bacterium gingive pyogenes; (3) Bacillus dentalis viridis; (4) Bacillus pulpæ pyogenes. The first of these organisms was found several times in a case of pyorrhea alveolaris, in the deposits around the teeth of a very dirty mouth. The second was found in the same case and also in a suppurating tooth pulp of a second person. The third variety was found in the superficial layers of a careous tooth. It produces a beautiful opalescent color in its growth. The fourth (bac. pulp. pyog.) was found in a gangrenous tooth and was proved pathogenic by inoculation.

Redness, swelling, abscess, diffuse suppuration, gangrene, general septic infection, with sometimes peritonitis or pleuritis, followed the inoculations—subcutaneous or inter-visceral—of these latter organisms. Mixed cultures are more dangerous than single germs in pure culture. Most disastrous of all are the bacteria of gangrenous pulps. Human saliva possesses most energetic toxic properties, as has been conclusively proven by fatality resulting from bites. Animals, especially white mice, almost invariably die after inoculation with sputum ; (1) Inoculation of rabbits and guinea-pigs, causes either death or the severest forms of septic infection ; (2) In one instance, where a mouse was inoculated with sputum, and died in eight hours, the writer found the Fränkel-Weichselbaum diplococcus of pneumonia disseminated through the blood ; this germ is nearly always present in the mouth, and all that is necessary for the development of pneumonia is exposure and reduction of the bodily temperature, when the germ takes on activity ; (3) In the presence of any morbid condition as gingivitis, wounds of the mucous membrane, dental caries, ulcerations, etc., the number of bacteria markedly increase. The diseases caused by pathogenic bacteria of the mouth Miller considers under six heads, formulated by Linsley as follows : (1) Infections caused by a solution of continuity in the mucous membrane, brought about by mechanical injuries (wounds, extractions, etc.); (2) Infections through the medium of gangrenous pulp ; these usually lead to the formation of abscess at the point of infection, but may cause secondary septicemia with fatal termination ; (3) Disturbances, produced by the absorption of poisonous products of the bacteria ; (4) Pulmonary disease, produced by the inspiration of particles of mucus, small pieces of tartar, etc., containing bacteria ; (5) Abnormal fermentative processes and other digestive disturbances caused by the swallowing of microbes and their poisonous products ; (6) Local infection of the soft structures of the buccial and pharyngeal cavities induced by mechanical irritation or constitutional debility ; (7) Possible infection through accumulation in the mouth of the germs of diphtheria, typhus, syphilis (?), tuberculosis, etc.

610 Main Street.

---

As Bromide of Strontium seems to be destined to displace the bromide of potassium, we would specially recommend our readers to insist on having the chemically pure salts (Paraf-Javal) dispensed, or the standard solutions (one gramme to the ounce), so as to avoid further accidents, as we learn that toxic effects have been caused by the dispensing of impure strontium salts, the poisonous barium being a concomitant of the strontium preparations of commerce.—*St. Louis Clinique.*

---

Hemorrhage.—Do not use styptics to control hemorrhage, and above all, do not use Monsel's solution (subsulphate of iron), as in case an operation is necessary it obscures the field of operation. The use of hot water is very much better (Prof. Keen).—*Coll. and Clin. Rec.*

